# Case report: ADULT syndrome: a rare case of congenital lacrimal duct abnormality

**DOI:** 10.3389/fgene.2023.1150613

**Published:** 2023-10-18

**Authors:** Jichao Zhou, Yuchen Wang, Yinghong Zhang, Debo You, Yi Wang

**Affiliations:** ^1^ Department of Ophthalmology, Peking University Third Hospital, Beijing Key Laboratory of Restoration of Damaged Ocular Nerve, Peking University Third Hospital, Beijing, China; ^2^ Department of Otolaryngology, Peking University Third Hospital, Beijing Key Laboratory of Restoration of Damaged Ocular Nerve, Peking University Third Hospital, Beijing, China

**Keywords:** TP63 gene, ADULT syndrome, congenital nasolacrimal duct obstruction, ectodermal dysplasia, dacryocystorhinostomy

## Abstract

Acro-dermato-ungual-lacrimal-tooth (ADULT) syndrome is a rare autosomal dominant inherited disease caused due to mutations in the *TP63* gene. More commonly, mutations in the *TP63* gene result in ectodermal dysplasia and/or orofacial cleft. ADULT syndrome is a type of ectoderm-related tissue dysplasia. This case report describes a patient with chronic tearing, congenital atresia, and obstruction of the lacrimal ducts, which are the main clinical manifestations of ADULT syndrome. This patient also presented with some clinical manifestations that were different from those of ADULT syndrome, namely, mild eyelid fusion and abnormal development of the fifth finger (a stiff fifth finger with camptodactyly that was shortened in length). The gene mutation in this patient was also at a site different from those usually reported in the literature. In this patient, c.518G > T resulted in p. G173V (accession number: NM_003722; exon4). We performed successful dacryocystorhinostomy and artificial lacrimal duct implantation. As shown above, we discussed the clinical characteristics and genetics of the disease in detail. In sharing this case, we aim to contribute to the current understanding of the genes and clinical manifestations of ADULT syndrome and to assist clinicians in the clinical diagnosis of *TP63* mutation-related diseases.

## Introduction

The presence of epiphora early in life is recognized as congenital nasolacrimal duct obstruction, with an incidence ranging from 5% to 20% (Petris and Liu, 2017). When considered as a single disease, obstruction is most often caused by a membrane at the end of the nasolacrimal duct called the valve of Hasner; this manifestation accounts for 73% of this disease, and 96% of the obstructions caused by the valve of Hasner resolve spontaneously (CASSADY, 1952). However, when congenital nasolacrimal duct obstruction or atresia is associated with dysplasia of other systemic organs, ectodermal dysplasia is often suspected. Acro-dermato-ungual-lacrimal-tooth (ADULT) syndrome is a common congenital disease associated with dysplasia of the lacrimal duct. It is a rare autosomal dominant genetic disease, first described in 1993, and is a type of ectodermal dysplasia. Other forms of ectodermal dysplasia include (Rinne et al., 2007) ankyloblepharon-ectodermal dysplasia-clefting syndrome (AEC), limb mammary syndrome (LMS), Rapp–Hodgkin syndrome (RHS), split-hand/split-foot malformation (SHFM), and ectrodactyly ectodermal dysplasia-cleft lip/palate syndrome (EEC). These ectodermal dysplasia types, including ADULT syndrome, are associated with mutations in the *TP63* gene (Avitan-Hersh et al., 2010; Prontera et al., 2011), which has a critical role in embryonic development, especially in the development of the limbs, ectodermal tissues, such as hair, skin, teeth, nails, and mammary glands. ADULT syndrome is characterized by sparse hair on the scalp and the axilla, lacrimal duct stenosis or atresia, onychodysplasia, hypodontia or the early loss of permanent teeth, athelia or hypoplastic nipples, and breast hypoplasia (Chan et al., 2004; Slavotinek et al., 2005). Some of the features of ADULT syndrome overlap with those of the other five types (mentioned above) of ectodermal dysplasia. The literature suggests that frequently mutated amino acids including R298Q, R298G, R243W, R227Q, P127L, R337Q, V114M and N6H, may be involved in ADULT syndrome (Slavotinek et al., 2005; Berk et al., 2012). In this report, we describe a patient with ADULT syndrome associated with a rare mutation of the *TP63* gene and atypical clinical features including mild symblepharon and a shortened, stiff fifth finger with camptodactyly.

## Case report

A 16-year-old Chinese female was referred to our hospital because of epiphora. The patient had experienced continuous and excessive production of tears without any stimuli since childhood, and there has been no significant change over the past 10 years. Over the last 4 years, she experienced sustained swelling, mild tenderness, and a detectable local mass on the right inner canthus that progressively enlarged. The ocular skin became dark and dull, and excessive tearing persisted. The patient’s personal and menstrual history were normal. Both the patient and her parents did not have any significant medical history, including history of carcinomas. Moreover, the patient’s mother and brother also presented with similar features including abnormal hair, nails, teeth, skin, and lacrimal ducts (Table 1). On clinical examination, the patient demonstrated the following features. The puncta were stenotic and bilaterally covered with a membrane. As a result, probing of the nasolacrimal duct was not possible on either side. Thus, aplasia of both lacrimal ducts with chronic tear production and the expansion of the obstructed lacrimal ducts leading to local enlargement around the lower lacrimal tubules were assumed. Furthermore, mild fused lower eyelids were evident.

**TABLE 1:** Clinical features noted in the affected family members.

		*TP63* mutation syndromes		
		Proband	Mother	Brother
Sex		Female	Female	Male
Age		16	50	5
Ectodermal	Teeth	√	√	√
	Skin	√	√	√
	Hair	√	√	√
	Nails	√	√	√
	Lacrimal ducts	√	√	√
	Breasts	√	√	√
	Sweat glands	-	-	-
Fused eyelids		√	-	-
Ectrodactyly		-	-	-
Ccenter lip and palate		-	-	-
Others		-	-	-

√ The clinical manifestations were observed, - The clinical manifestations were not observed.

**FIGURE 1:**
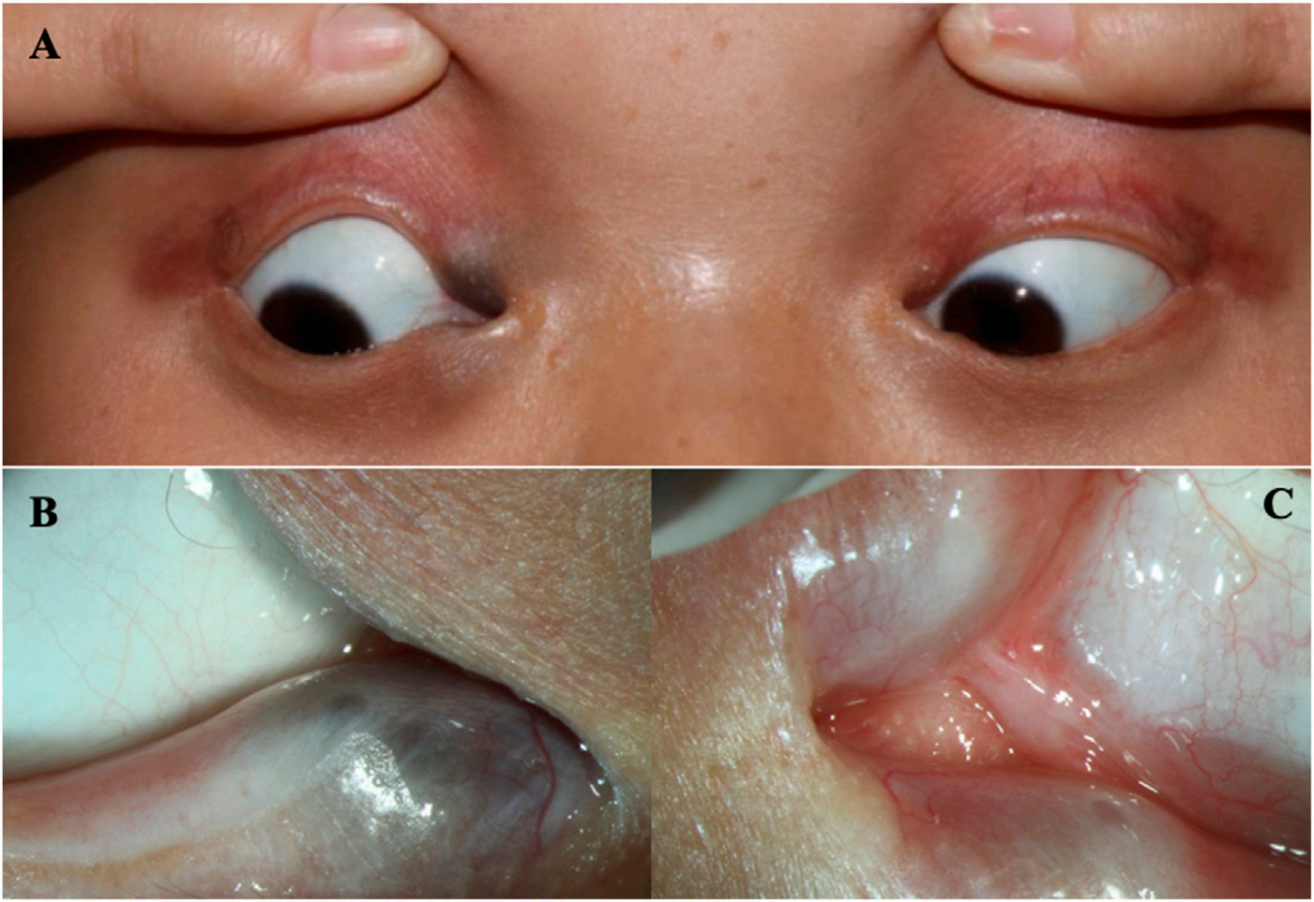
The eyes of the patient **(A)** The clinical appearance of the eyes. **(B)** The enlargement around the lower lacrimal tubules. **(C)** Absence of the upper puncta and closure of lower puncta.

**FIGURE 2:**
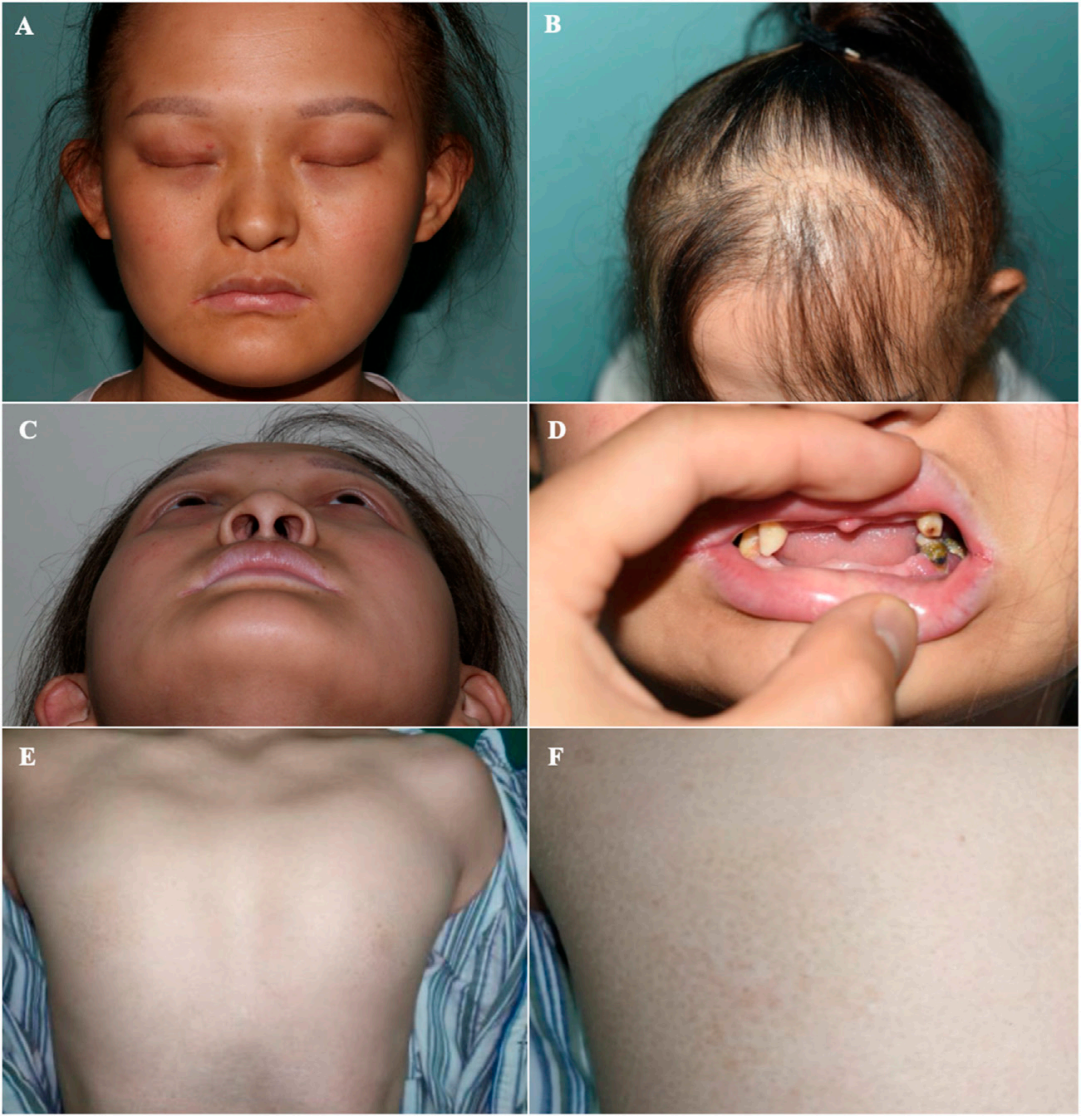
Orofacial and mammary glands’ features of the patient **(A)** Facial phenotype of the proband with sparse eyebrows with tattooing, absent eyelashes, small ears, and a hooked nose. **(B)** Sparse brown hair, especially in the front of her scalp. **(C)** A hollow facial appearance. **(D)** Dental abnormalities including hypodontia or oligodontia and conically shaped teeth. **(E, F)** Absent mammary glands with bilateral hypoplastic nipples.

**FIGURE 3:**
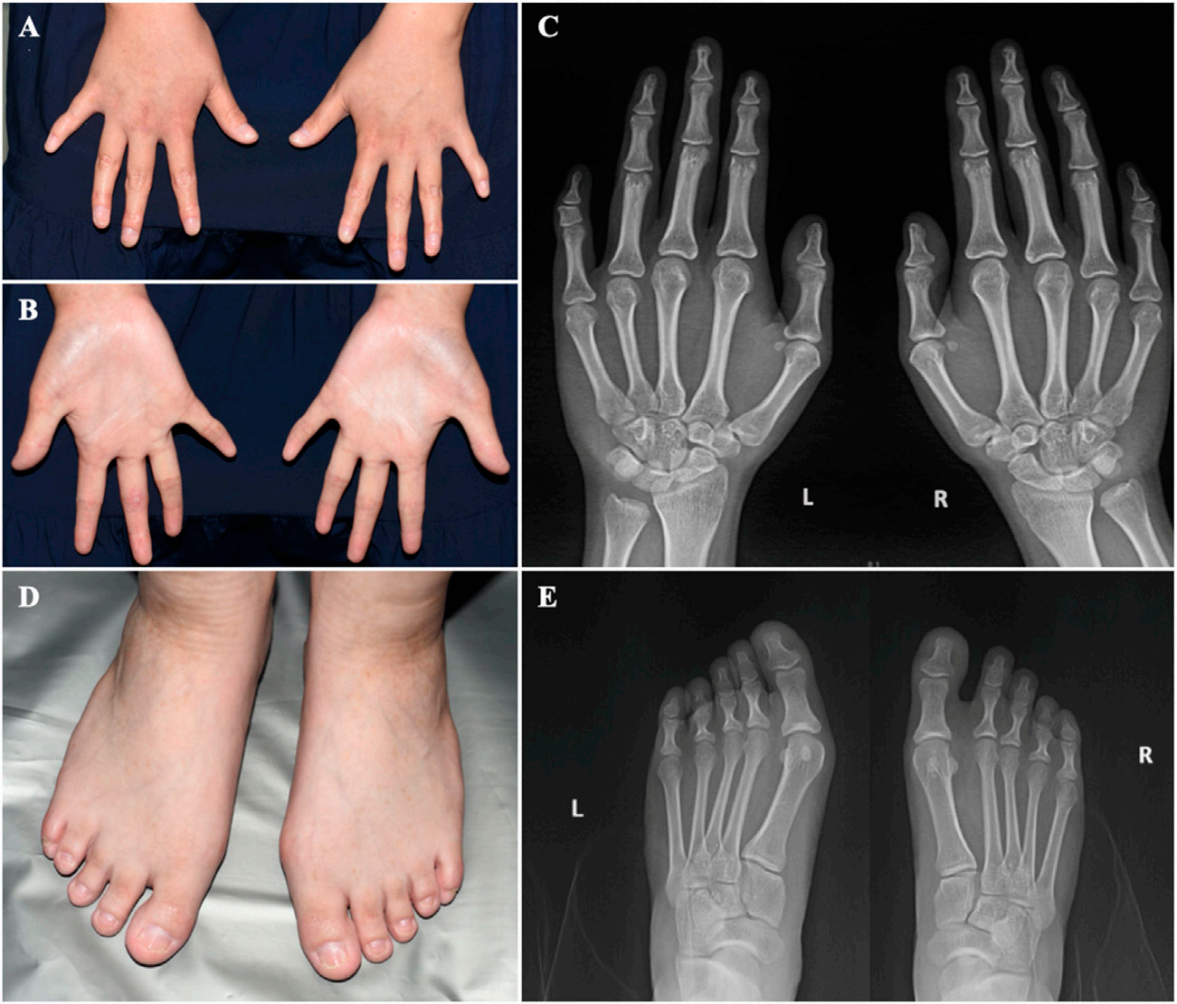
The hands and feet of the patient **(A)** Bilateral clinodactyly of the fifth finger. **(B)** Palmar hyperlinearity. **(C)** Radiograph of the hands showing clinodactyly of the fifth fingers. **(D)** Dystrophic nail plates and horizontal grooves along the length of the nails. **(E)** Radiograph of the feet.

On physical examination, the following features were observed. (1) Skin: sweaty, pale, and without freckles; (2) Hair: brown and sparse, especially in the front of her scalp; (3) Oral cavity: conical teeth and hypodontia or oligodontia; (4) Nose and ears: small ears and a hooked nose; (5) Mammary glands: absent and bilateral hypoplastic nipples; (6) Hands: brachydactyly, which was most prominent in her fifth fingers, and bilateral fifth finger clinodactyly and camptodactyly; (7) Nails: discolored and irregularly shaped, with short and dystrophic nail plates and horizontal grooves along the length of the nails (Figures 1–3).

**FIGURE 4:**
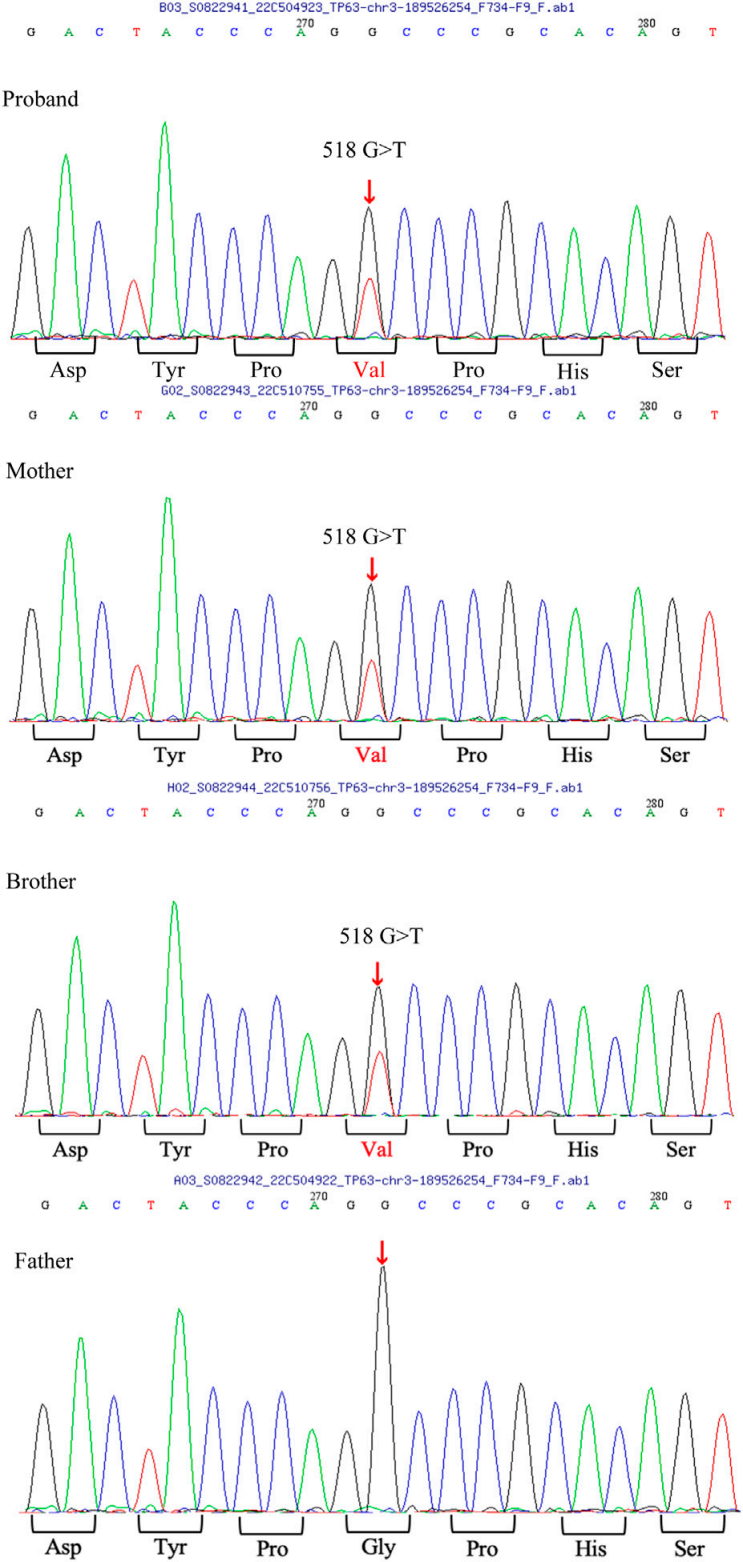
*TP63* mutation analysis A heterozygous G>T transition at cDNA position 518 of the *TP63* gene is found in the patient, as well as in her mother and brother.

**FIGURE 5:**
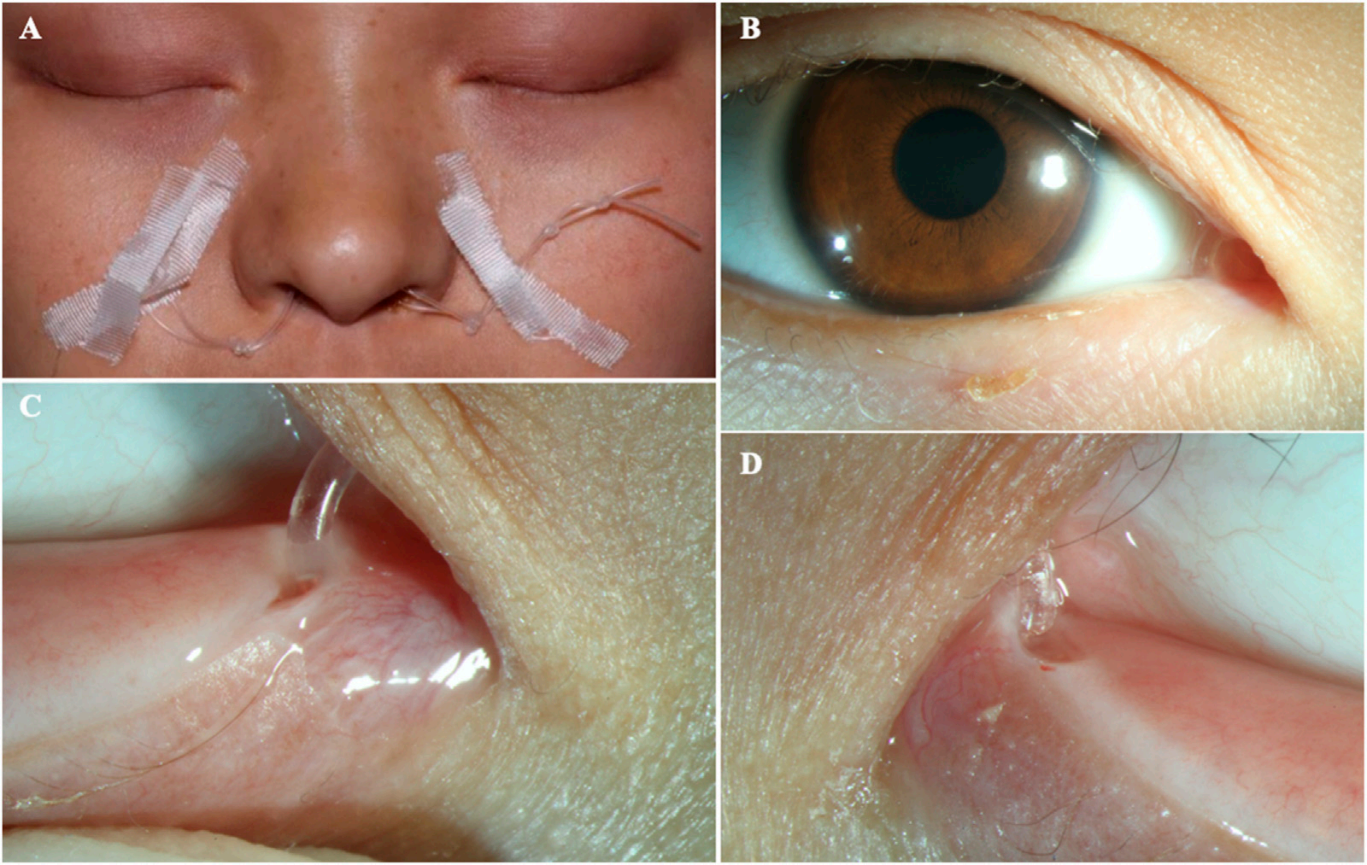
The patient after surgery **(A–B)** The ocular appearance after surgery. **(C)** The artificial nasolacrimal duct. **(D)** The close-up appearance of the opened punctum.

After obtaining written informed consent from the patient and her parents, peripheral blood samples were collected. Whole-exome sequencing was performed to screen for candidate mutations. Called mutations were validated using Sanger sequencing. We identified a heterozygous G>T transition at cDNA position 518 of *TP63* (accession number: NM_003722; exon4; OMIM number 103285) (Figure 4). This mutation is predicted to result in amino acid substitution p. G173V, and its functional effect, analyzed by two prediction tools (SIFT and PolyPhen), was predicted to be deleterious, thus supporting its pathogenicity. In the literature, mutation at cDNA position 518 has been previously reported (Chan et al., 2004) in a patient with ADULT syndrome, with cleft lip and palate. However, the transverse changes of the amino acids in that case were different from those in our patient. To the best of our knowledge, this is the first reported clinical variation of ADULT syndrome with a rare mutation, distinguished by the clinical manifestation of symblepharon and camptodactyly. After confirming the diagnosis, the patient’s chronic epiphora was addressed via binocular dacryocystorhinostomy under general anesthesia, during which artificial tear ducts were placed to drain the tears, and the enlarged lacrimal duct was removed. The surgery was successful, and the patient showed no lacrimal abnormalities on follow-up (Figure 5).

## Discussion

The *TP63* gene is highly expressed in the nuclei of the basal cells of the skin, cervix, tongue, mucosa, esophagus, mammary glands, prostate, and urothelium (Rinne et al., 2007). A crucial transcriptional regulator factor, *p63* is often expressed in the epithelial and mesenchymal tissues (Otsuki et al., 2020). It is expressed very early during embryogenesis and epidermal development and plays an essential role in the induction of the ectoderm and the orofacial, limb, and epidermal stratification processes. Moreover, the expression of P-cadherin, which is regulated by *p63*, acts as a critical regulator of hair development. Previous studies have confirmed that the normal expression of *p63* can inhibit the terminal differentiation of keratinocytes, which contributes to maintaining the proliferative potential of the basal cell layer and promoting its formation and integrity (Avitan-Hersh et al., 2010). In view of this, *in vitro* experiments performed in 1999 confirmed that *TP63* gene knockout mice developed ectodermal developmental defects, such as limb defects and the loss of the prostate, mammary glands, epidermis, and other related tissues (Wang et al., 2009). These features were representative of defective ectodermal stem cells and were consistent with the physiological functions of the *TP63* gene (Rinne et al., 2007). Since then, syndromes associated with *TP63* mutations have been recognized in multiple reports, including the AEC, LMS, ADULT, RHS, SHFM, and EEC. The specific clinical manifestations of these six diseases are summarized in Table 2 (Duijf et al., 2002; Rinne et al., 2006a; Rinne et al., 2006b; Rinne et al., 2007; Otsuki et al., 2016).

**TABLE 2:** Clinical features in six overlapping syndromes.

		*TP63* mutation syndromes							
		*Our case*		AEC	LMS	ADULT	RHS	SHFM	EEC
Ectodermal	*Teeth*		√	+++	++	+++	+	-	+++
	*Skin*		√	+++	-	+++	+	-	+++
	*Hair*		√	+++	-	+++	+	-	+++
	*Nails*		√	+++	+++	+++	+	-	+++
	*Lacrimal ducts*		√	+++	+++	+++	+	-	+++
	*Breasts*		√	+++	+++	+	-	-	+
	*Sweat glands*		-	+++	++	-	+++	-	+
Fused eyelids			√	+++	-	-	-	-	-
Ectrodactyly			-	-	+++	-	++	+++	+++
Ccenter lip and palate			-	++	++	-	+++	-	+++
Others			-	Hearing impairment++	-	-	-	-	Hearing impairment+
				Genito-urinary++					Urinary+

√ The clinical manifestations were observed. - The clinical manifestations were not observed.

^+++^Frequently observed in >50% of patients, ++ observed in 30%–50% of patients, + occasionally observed in 30% of patients, and rarely or never observed.

AEC, ankyloblepharon-ectodermal dysplasia-ccentering syndrome; LMS, limb mammary syndrome; ADULT, Acro-dermato-ungual-lacrimal-tooth syndrome; RHS, Rapp–Hodgkin syndrome; SHFM, split-hand/split-foot malformation; EEC, ectrodactyly ectodermal dysplasia-ccenter lip/palate syndrome.

Our patient presented with clinical features of ectodermal dysplasia, with sparse hair, dystrophic nails, small teeth, oligodontia (11 teeth left), lacrimal duct stenosis, and hypoplastic nipples. These clinical features are observed in different syndromes. For EEC, orofacial cleft and ectrodactyly are typical manifestations. In contrast, cleft lip and palate are typically not detected in ADULT syndrome. Hence, this feature can be used to distinguish EEC from ADULT syndrome. On the contrary, it is difficult to distinguish between LMS and ADULT syndrome. LMS also manifests as a form of ectodermal dysplasia with oligodontia, lacrimal atresia, and nail dystrophy, in addition to abnormal development of the mammary glands and hypoplastic nipples. These findings may render a definitive diagnosis challenging. However, most patients with ADULT syndrome present with ectrosyndactylia and hair and skin abnormalities that have not been reported in LMS, and thus, these features may assist with diagnosis. Moreover, our patient had mild symblepharon that can also be observed in AEC, as well as ectodermal-related manifestations, which led us to suspect that the patient may have AEC. However, according to a literature review (Slavotinek et al., 2005; Kawasaki de Araujo et al., 2017), cleft lip and palate are characteristic of most patients with AEC, and more than half of all patients with AEC have hearing impairment and urinary system diseases, which were not consistent with the presentation of our patient. Furthermore, AEC is not typically associated with abnormal limb development. Hence, our patient, with her shortened fifth fingers, was suspected to have ADULT syndrome. Clinically, the six diseases mentioned above share some common manifestations. However, they have different gene inheritance patterns and also some relatively unique features (Rinne et al., 2006a; Rinne et al., 2006b; Rinne et al., 2007).

The literature suggests that the most common site of mutation of the *TP63* gene is the DNA binding region, due to a missense point mutation, resulting in the substitution of arginine 298 by glycine or glutamine. *In vitro* experiments (Chan et al., 2004) confirmed that R298 is not adjacent to the DNA binding domain. Therefore, the mutation of amino acid 298 does not lead to any adverse effects, but results in high transactivation activities of ΔN-p63γ, which may be 25% higher than those of wild-type *p63* (Duijf et al., 2002). Missense point mutations in exon 3 can result in the substitution of p. N6H (asparagine to histidine), ultimately resulting in ADULT syndrome. N6H is in the upstream region of the DNA domain of *p63* and is only contained in the *p63* subtype of the transactivation domain of this protein, which does not affect the activity of the *p63* DNA binding domain (DBD) (Slavotinek et al., 2005). However, the above mutations are significantly different from those in EEC. For example, R298G and R298Q increase the activity of *p63*, while N6H, which is outside the functional domain of *p63*, does not affect the expression of *p63*. However, missense mutations in EEC are likely to result in the loss of DNA binding and impaired transactivation activities (Amiel et al., 2001). As a result, an essential difference is detectable at the genetic level, and this can be used to exclude ADULT syndrome.

In our patient, the mutation site was *p63*, p. G173V, which was consistent with a previously reported mutation. Monti et al. (2013) detected the transactivation abnormality and interfering ability of this mutant protein in yeast and mammalian cells and quantified the protein functional changes after mutation. Monti et al. detected wild-type and *p63* p. G173V protein changes by inducing galactosyl-dependent protein expression in yeast through the inducible GAL1, 10 promoter and revealed that the mutation resulted in a 20% reduction in transactivation activities at relatively high galactose concentrations (0.128%). In mammalian cells, the mutation p. G173V retains partial transactivation activity. For example, p. G173V mutants show a high residual transactivation potential on the P21, MDM2, PUMA, and BAX targets, which are regulated by *p63*, and are involved in the regulation of the cell cycle, protein stability, apoptosis, and epithelial cells.

PERP and COL18A1 are well-known *p63*-regulated genes involved in skin and epithelial development. As for the interfering ability, the p. G173V mutant clearly interferes only with PERP and COL18A1 targets to lower the transactivation ability compared to wild-types. Considering the corresponding structure and function, the region corresponding to amino acid 173 protrudes on the surface of the protein, so that the protein’s functional structure does not change. The reason for these functional changes may be that amino acid 173 is close to the N-terminus of the DBD, which is involved in the recruitment and assembly of tetramer proteins that affects the machinery of transcription proteins. Meanwhile, amino acid 173 is located in the proline-rich region of the C-terminus, which is important for the structural integrity and apoptosis-inducing function of the transcription protein. Thus, the mutation induces changes in the transactivation and interference ability of the protein.

Different amino acid mutations result in different transactivation abnormalities and interfering abilities, as well as different clinical phenotypes. However, it can be seen from the above discussion that the clinical manifestations of the six *TP63*-related syndromes overlapped greatly. One amino acid mutation site can cause more than one syndrome. For example, as reported by Avitan-Hersh et al. (2010), the mutated amino acid p. R243W was previously reported to be associated with EEC and LMS. Brunner and Van Bokhoven (2002) also reported a patient with the ADULT syndrome phenotype, but her mutated amino acid (R227Q) was previously related to EEC and LMS. As such, in addition to the overlapping clinical phenotypes, the mutations affecting the amino acids in the *TP63* gene also have a certain crossover potential. In other words, even subtle phenotypic differences can represent diversities at the molecular level. For example, the ADULT syndrome mutations can occur in exons 3, 4, 6, or 8, while LMS mutations can occur in exons 4, 13, and 14 (Rinne et al., 2007), and EEC mutations can occur in exon 5–8, 13, or 14 (Rinne et al., 2007; Whittington et al., 2016). For this reason, (Monti et al., 2013), proposed that clinical conditions should be integrated with the functional parameters of *p63* mutated proteins, such as the transactivation ability of target genes involved in specific developmental pathways and the interaction and interference ability of mutant isomers, for better genetic diagnosis and differentiation of the overlapping clinical phenotypes (Serra et al., 2011).

At present, there is no treatment for conditions caused by *TP63* mutations. However, we can focus on ensuring an accurate diagnosis by differentiating the different types of diseases with *TP63* mutations and further improve the accuracy of genetic counseling through gene analysis. In the future, we hope we can assist patients in making reproductive plans and offer therapeutic means to cure the genetic disease from the embryonic stage through the classification of gene variants, such as gain-functional mutations or loss-functional mutations (van Zelst-Stams and van Steensel, 2009; Monti et al., 2013).

## Conclusion

In summary, we reported a case of ADULT syndrome caused by a rare amino acid mutation with a rare clinical phenotype, including eyelid fusion and abnormal development of the fifth finger. These findings add to our current understanding of ADULT syndrome and other *TP63*-related diseases.

All relevant information was explained to the patient and his mother, and written informed consent was obtained from the patient and his mother for publication of this case report and accompanying images.

## Data Availability

The original contributions presented in the study are included in the article/Supplementary Material, further inquiries can be directed to the corresponding author.
